# Collaborative community based care for people and their families living with schizophrenia in India: protocol for a randomised controlled trial

**DOI:** 10.1186/1745-6215-12-12

**Published:** 2011-01-13

**Authors:** Sudipto Chatterjee, Morven Leese, Mirja Koschorke, Paul McCrone, Smita Naik, Sujit John, Hamid Dabholkar, Kimberley Goldsmith, Madhumitha Balaji, Mathew Varghese, Rangaswamy Thara, Vikram Patel, Graham Thornicroft

**Affiliations:** 1 Sangath, Porvorim, Goa, India; 2 Health Service and Population Research Department, Institute of Psychiatry, King's College London, UK; 3 London School of Hygiene & Tropical Medicine, UK; 4 Schizophrenia Research Foundation, Chennai, India; 5 Parivartan, Satara, India; 6 National Institute of Mental Health and Neurosciences, Bangalore, India; 7 Jawaharlal Nehru Medical College, Karnataka, India; 8 Nirmittee, Satara, India

## Abstract

**Background:**

There is a large treatment gap with few community services for people with schizophrenia in low income countries largely due to the shortage of specialist mental healthcare human resources. Community based rehabilitation (CBR), involving lay health workers, has been shown to be feasible, acceptable and more effective than routine care for people with schizophrenia in observational studies. The aim of this study is to evaluate whether a lay health worker led, Collaborative Community Based Care (CCBC) intervention, combined with usual Facility Based Care (FBC), is superior to FBC alone in improving outcomes for people with schizophrenia and their caregivers in India.

**Methods/Design:**

This trial is a multi-site, parallel group randomised controlled trial design in India.

The trial will be conducted concurrently at three sites in India where persons with schizophrenia will be screened for eligibility and recruited after providing informed consent. Trial participants will be randomly allocated in a 2:1 ratio to the CCBC+FBC and FBC arms respectively using an allocation sequence pre-prepared through the use of permuted blocks, stratified within site. The structured CCBC intervention will be delivered by trained lay community health workers (CHWs) working together with the treating Psychiatrist. We aim to recruit 282 persons with schizophrenia. The primary outcomes are reduction in severity of symptoms of schizophrenia and disability at 12 months. The study will be conducted according to good ethical practice, data analysis and reporting guidelines.

**Discussion:**

If the additional CCBC intervention delivered by front line CHWs is demonstrated to be effective and cost-effective in comparison to usually available care, this intervention can be scaled up to expand coverage and improve outcomes for persons with schizophrenia and their caregivers in low income countries.

**Trial registration:**

The trial is registered with the International Society for the Registration of Clinical Trials and the allocated unique ID number is ISRCTN 56877013.

## Background

Schizophrenia is a severe mental disorder that usually has an onset in early adulthood and is often associated with persistent or relapsing symptoms and a range of other adverse outcomes [1,2]. Even though a low prevalence disorder, schizophrenia contributes an estimated 1.1% of the total Disability Adjusted Life Years (DALY's) and 9.5% of the total DALY's attributable to the neuropsychiatric disorders [3] in South East Asia making it a priority public health concern for the region. There is international consensus that care for people with schizophrenia should largely be delivered in community settings for the best outcomes[4]. While in high resource countries, community care is the norm, in low income countries, the availability of community services is the exception[5]. Thus, for the majority of persons with schizophrenia and their caregivers in low income countries, including in India, there is little access to any care [6]. Addressing this 'treatment gap' by scaling up accessible, acceptable and effective community based services for persons with schizophrenia is an urgent public health and ethical priority in countries like India[7].

Scaling up of community services in low income countries faces challenges like the paucity of specialist resources and the lack of evidence around the effectiveness, costs and processes involved in delivering community based interventions. One method that specifically addresses the challenges of the lack of specialist resources, accessibility and inequity in care provision is the Community Based Rehabilitation (CBR) strategy for persons with disabilities- a health and social intervention that is largely implemented by appropriately trained, lay persons within community settings[8]. In addition, the CBR method has a strong focus on empowerment of service users, social inclusion, livelihood support and equalising of opportunities for people with disabilities; all of these are of particular relevance to persons with schizophrenia. A non-randomised controlled study from rural India showed that CBR using trained lay community workers was feasible and improved outcomes for people with schizophrenia, compared with routine outpatient care [9]. A more recent study from the same site has described the impact of specific components of the complex intervention on long-term outcomes in people with schizophrenia[10]. These encouraging results need to be rigorously tested through a randomized controlled trial. If demonstrated to be clinically effective and affordable in comparison to usually available care (i.e. facility based care) alone, such interventions can be scaled up expand coverage and improve outcomes of persons with schizophrenia and their caregivers in low income countries by using available and low cost human resources.

### Objectives

The objectives of the trial are: firstly, to test the hypotheses that usual, Facility-Based Care (FBC), when combined with a Collaborative Community-Based Care intervention (CCBC), will be superior to Facility-Based Care alone in improving a range of outcomes in people with schizophrenia and their caregivers and, secondly, to determine if the CCBC intervention is cost effective in India.

The primary objectives of the trial are to determine whether the FBC+CCBC intervention will be superior to FBC alone in:

i) reducing symptoms of schizophrenia at 12 months, and

ii) reducing disability related to schizophrenia over 12 months.

In *persons with schizophrenia*, the secondary objectives are to determine whether, FBC+CCBC will be more effective than FBC alone, in:

• improving adherence to antipsychotic treatment

• reducing experiences of stigma and discrimination

For *caregivers of people with schizophrenia*, the secondary objectives are to determine

whether, FBC+ CCBC will be superior to FBC alone, in:

• improving their knowledge and attitudes about the illness

• reducing their burden of caring

• reducing experiences of stigma and discrimination.

The health economic objective is to compare the costs and assess the cost-effectiveness and cost-utility of the FBC+ CCBC intervention compared to FBC alone.

The COPSI trial is a collaborative effort between multiple institutions and individual Psychiatrists at the Indian sites. The lead institution is the Institute of Psychiatry (IoP), Kings' College, London, while the partner organizations are the London School of Hygiene and Tropical Medicine (LSHTM), Sangath in Goa, the Schizophrenia Research Foundation (SCARF) in Chennai, the National Institute of Mental Health and Neuroscience (NIMHANS) in Bangalore and Parivartan and Nirmittee in Satara. In Goa and in Satara, the individual collaborators are Psychiatrists working in the private sector.

## Methods/Design

### Study design

Effectiveness, parallel group, randomised controlled trial design with unequal allocation of participants between arms. The two arms of the study (allocation ration 2:1) will compare people with schizophrenia allocated to receive: (i) FBC+ CCBC, or (ii) FBC only, with follow up over 12 months.

### Setting

The trial will be conducted in three sites in India- in four rural blocks or sub-districts in the southern Indian state of Tamil Nadu and in two sites in western India, - across the state of Goa and in Satara in the state of Maharashtra. The relevant details of the sites are summarized in Table 1. These three sites have been chosen to reflect a diversity of health system contexts, to strengthen the generalisability of the study findings and to meet trial recruitment targets within the allocated timeframe.

**Table 1:** The characteristics of the COPSI trial sites.

Site	Tamil Nadu	Goa	Satara
**Collaborators**	Schizophrenia Research Foundation http://www.scarf.org	Consulting Psychiatrists in private sector	Consulting Psychiatrists in private sector and 'Parivartan' and 'Nirmittee'

**Type of service provider**	Non Government Organization (NGO)	Private sector individual practices	Mixed model- combination of private consulting Psychiatrists and NGO's working in tandem

**Catchment area description**	Population coverage of approximately 500,000.	Population coverage of approximately 1 million.	Population coverage of approximately 2.0 million from nearby urban and rural areas
	Participants will be from 235 villages in Kanchepuram district of Tamil Nadu.	Referrals treated from all parts of Goa.	Referrals are from the surrounding urban and rural areas of western Maharashtra
	People with schizophrenia mainly from rural settings.	People with schizophrenia mainly from urban and peri-urban settings.	People with schizophrenia from both rural and urban settings
	Majority belong to lower socioeconomic section of the community.	Predominantly middle and upper socio-economic status population who can afford private care.	Wide range of social classes represented.

**Usually available treatments at facilities**	*Services available:* Out- patient care once every 2 weeks at each of the community clinic centres.	*Services available:* All collaborators are solo practitioners	*Services available:* Out-patient care 6 days a week in designated clinics
	1 Psychiatrist, 1 Psychiatric Social Worker and 1 trained Assistant available at each centre during the clinic.	Out-patient care 6 days a week in designated urban clinics In-patient care provided through liaison with private hospitals	IP care: In multi specialty hospital with registered private psychiatric unit for 20 psychiatric beds.
	*Intervention:* Main treatments are antipsychotic and other psychotropic medications and brief counselling for families and persons with schizophrenia.	*Intervention:* Main treatments used are anti-psychotic and other psychotropic medications with brief advice and counselling for families and persons with schizophrenia.	*Intervention:* Main treatments used are anti-psychotic and other psychotropic medications with brief advice and counselling for families and persons with schizophrenia
	*Description of usual care process:* Consultation lasts for about 20 minutes and usually includes family members.	*Description of usual care process:* Consultations last about 30 minutes and usually include family members.	*Description of usual care process:* Consultations last about 30 minutes and usually include family members.
	*Costs of medicines:* Free medications are given to all persons with schizophrenia attending the community clinics.	*Costs of medicines:* Medicines are purchased from pharmacies entirely through out of pocket expenses	*Costs of medicines* Medicines are purchased from pharmacies entirely through out of pocket expenses

**Whether any community care is already available**	No community care available	No community care available	No community care available

**Number of inpatient beds**	Nil- referrals to existing hospitals when necessary for acute care	On an as needed basis in private hospitals	20

**Average number of total OP attenders/week**	50-60	200	400-500

**Average number of patients/week meeting eligibility criteria**	6-8	5-6	10-12

### Description of the Interventions

#### The collaborative community based care (CCBC) intervention

The CCBC intervention is underpinned by three key principles:

• firstly, the intervention is planned and implemented for each participant in close collaboration between the person with schizophrenia, the primary care giver(s) and the treatment team members (comprising the lay CHW, a supervising Psychiatric Social Worker and the treating Psychiatrist);

• secondly that the intervention is provided in a flexible manner that reflects the unique needs of the person with schizophrenia and their caregivers and;

• thirdly, that the intervention will promote respect, autonomy and dignity for the person with schizophrenia and caregivers.

The inclusion of the specific treatment components within the treatment package have been guided by:

• evidence that the treatment is locally feasible, acceptable and potentially of low cost;

• evidence for the effectiveness of the treatment in low and middle income settings and;

• evidence that it can be delivered by lay health workers

The CCBC intervention will be coordinated and delivered by lay CHWs who have completed at least 10 years of schooling and have been systematically trained at each site over 6 weeks. The training is based on the intervention manual that follows a modular structure covering various aspects of the illness, specific components of the intervention and the operational requirements of the trial related documentation and supervision http://www.sangath.com/details.php?nav_id=60. The CHWs will be closely supervised at each site by Psychiatric Social Workers working as designated Intervention Coordinators. They will coordinate the overall delivery of the intervention and assure the quality and fidelity of the intervention at the site. The treating Psychiatrists will provide clinical leadership for the community care teams and ongoing supervision to maintain safety and quality standards. Overall, the continued supervision of the CHWs through joint onsite visits, weekly group meetings and in scheduled meetings with the Psychiatrist is an essential component of the CCBC intervention.

The treatments included in the intervention package are:

• *structured psycho-educational information *about various issues related to the understanding and proactive management of the illness for the participant and the care givers as well as specific efforts to identify and address experiences of stigma and discrimination

• *adherence management *strategies to reduce the rates of non-adherence to treatments

• *health promotion strategies *to improve the physical health status of people with schizophrenia

• Specific *rehabilitation strategies *to improve the personal, social and vocational functioning of participants

• Linkage to *self help groups *and other methods of user led support

• Developing *networks with community agencies *to address social problems in the family like poverty or interpersonal disputes and facilitate a supportive framework for participants to seek employment and access to social and legal benefits

The intervention is delivered in three phases. In the initial, 3 month *intensive engagement phase*, the key tasks will include developing a positive therapeutic alliance with the person with schizophrenia and care givers, conducting the needs assessment and generating the collaborative individual treatment plan. There will also be a focus on identifying and addressing familial social difficulties that have an adverse impact on the person with schizophrenia, adherence management and structured psycho-education sessions. We anticipate that CHWs will conduct 6-8 home based (or other convenient location nominated by the person and care givers) sessions during this phase.

In the second, *stabilization phase *during months 4-7, the CHWs will conduct a total of 6-8 fortnightly sessions. In this phase, the focus will be on addressing the unmet or partially met needs from the first phase, reinforce psycho-education and adherence, and introduce other components like health promotion, rehabilitation needs assessment and interventions (for example, improving activities of daily living skills and social skills training), and improving social interactions within and outside of the family.

In the final, *maintenance phase *during months 8-12, the CHWs will conduct 6 sessions. In this phase we intend to reinforce the progress made, discuss strategies to deal with distressing symptoms, discuss ways of dealing with experiences of stigma and discrimination, focus on restoration of social and economic roles, generate a relapse prevention plan and address remaining unmet needs, to the extent possible before a carefully planned termination.

In total, we anticipate that each participant in the CCBC arm will receive a maximum of 22 planned contacts during the 12 months of the intervention, and that each CHW will carry a maximum case load of 25 people with schizophrenia at any one time. In evaluating the intervention, we have set an a priori definition of 'minimum adequate' as being 12 sessions of the CCBC sessions delivered over 12 months.

All informational materials meant for the use of participants and caregivers has been systematically translated into the five regional languages used across the trial sites. The intervention has been systematically piloted at each of the sites. Based on the results of the field testing, the final content and process of the phase specific delivery of the intervention has been manualised, as mentioned earlier.

#### Facility based Care (FBC)

'Facility Based Care' is the care usually provided by mental health practitioners for persons with schizophrenia and their families. In two of the sites i.e. Goa and Satara, Psychiatrists in the private sector are the primary care providers with trained assistants recording essential socio demographic details in some of these clinics. In rural Tamil Nadu, a team of three persons are involved in the clinics. A trained assistant records essential socio demographic details of all persons who come to the clinic. A brief clinical history is then elicited by the Psychiatric Social worker (PSW) who then presents the findings to the clinic Psychiatrist for detailed assessment and treatment planning. In Goa and in Satara, the Psychiatrists are the first point of contact and, for the most part, conduct all necessary procedures themselves. Across all settings, consultations last from 15-45 minutes; persons with schizophrenia presenting for the first time are reviewed once or twice a month while those who are clinically stable are reviewed more flexibly- once a month- 3 months. Almost all persons with schizophrenia in these facilities are prescribed psychotropic medications with the newer antipsychotic medications being preferred (as in India, these newer drugs are cost comparable with the older antipsychotic drugs) while the use of antidepressants, anticholinergic, sedatives and modified Electroconvulsive treatment (ECT) is based on individual clinical needs. All psychiatrists also provide specific information about the illness, encourage adherence and discuss other specific concerns that the person with schizophrenia or their family members have; all of these are provided on an 'as needed' basis. The salient difference between the two arms is that in the CCBC arm, a complex psychosocial intervention, tailored towards individual needs, will be delivered in an accessible (home based) and structured manner by a dedicated CHW. There will be no guidelines or protocols made available to Psychiatrists for the medical treatment of participants as this is more representative of usual care in India. FBC will continue to be available to participants in both study arms and treating Psychiatrists will be unblinded while providing care for participants.

### Selection of participants

COPSI is an effectiveness trial and to improve the generalisability of the study results, we will:

• recruit persons with schizophrenia into the trial from those who present to routine, 'real life' treatment settings and

• include those persons with schizophrenia for whom community based care is justified even in low resource countries.

Thus, the inclusion criteria for participating in the trial are:

• Trial participants must be between the ages of 16-60 years

• Have a primary diagnosis of schizophrenia as per ICD-10 DCR criteria [11]

• Have had an illness duration of at least 12 months and an overall moderate severity of the illness based on the Clinical Global Impression-Schizophrenia(CGI-SCH) scale [12] rating. This reflects the threshold at which additional community care is warranted in resource constrained settings where community based care can realistically be offered only for those with high needs.

• Be residing within the study catchment area for the next 12 months.

In the Tamil Nadu site, participants will be selected from those identified through a community survey in the study area and will then be referred to community clinics for assessment by the Psychiatrists. In Goa and Satara, participants will be approached for recruitment from the clinical practices of the collaborating Psychiatrists. In these settings, we anticipate two pathways for recruitment: (a) people with schizophrenia from within the existing caseloads at each of the sites who meet inclusion criteria; and (b) people with schizophrenia who have presented for care for the first time and meet all inclusion criteria.

### The flow of participants in the trial

The collaborating Psychiatrists will identify all persons with a primary diagnosis of ICD 10-DCR schizophrenia in their setting by using a checklist. All persons with schizophrenia will be assigned a unique identification number and the Psychiatrist will fill out an initial screening form to confirm eligibility and record other basic socio-demographic and clinical details. From this 'universe' of persons with schizophrenia, for those who meet all inclusion criteria, the Psychiatrists will provide a brief overview of the trial together with a standard flier outlining the purpose of the trial (that is translated to all relevant local languages) and ask the person and the caregiver (s) whether they are interested in participating in the trial. If the person or the caregiver does not agree to proceed further, the Psychiatrist will briefly record their reasons for their not agreeing, whenever possible. For those who have refused, the Psychiatrist has the option of revisiting their participation during the course of the recruitment period. If both are interested to continue, the Psychiatrist will then fill out additional clinical details and take written (for non literate persons, their left thumb impression will be taken) assent for the independent consent procedure.

Subsequently, an independent, trained researcher will conduct the informed consent interview. The consent procedure has been specially designed to provide adequate information about the trial in a manner that is matched to the literacy levels of the participant and caregiver and to minimize the cognitive difficulties that persons with schizophrenia commonly face in processing information for making an informed decision[13]. After the procedure, the participant and the caregivers make an informed choice to participate or not. At this stage, the researcher will record the reasons for participation or refusal, whenever possible. Once informed consent has been obtained, trained researchers will complete the baseline quantitative assessments for both the participant and the caregiver(s) who are then assigned a unique trial ID.

From this overall sample of participants, a purposively selected sub sample of 12 participant and their caregivers will be chosen at each of the three sites for the additional baseline qualitative interviews. Once all baseline assessments have been completed, participants will be randomly assigned to either of the 2 arms of the study for the next 12 months.

**Figure 1:**
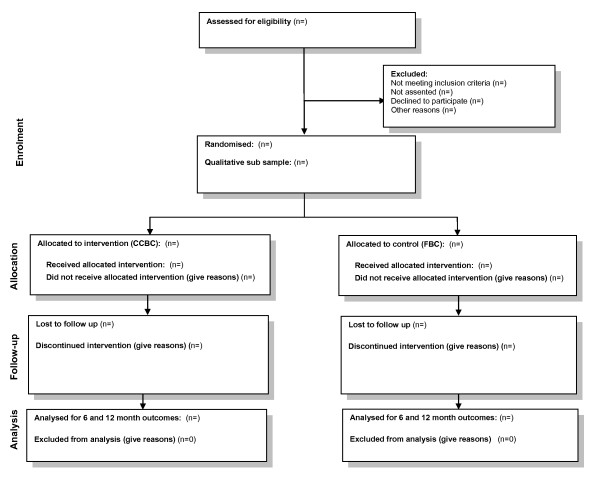
Flow of participants during the conduct of the trial

The identification, registration and subsequent flow of participants in the trial will be governed by the trial standard operational protocol (SOP) at all sites. This will be monitored closely using a set of process indicators and any deviations from the protocol and the reasons for the same will be carefully recorded. The flow of participants during the conduct of the trial, illustrated in Figure 1, will be monitored and reported in compliance with the recent CONSORT recommendations[14].

### Randomisation

#### Generating the allocation list

Three lists have been generated independently by the Trial Statistician in London, one for each site, and transmitted to the site Data Managers prior to the commencement of the recruitment for the trial. The pre determined allocation sequence cannot be altered during the recruitment of participants in the trial.

#### Allocation concealment

The site Data Managers will be entrusted with keeping the allocation list secure (through password protected files). The matching of the study numbers of the recruited participants with their randomization numbers and the concealment of randomization from interviewers will also be managed by the central Data Manager to ensure that no one has access to the allocation list.

#### Implementation

For logistic reasons, the site Data Managers will be responsible for assigning the unique trial ID for each randomized participant in the trial; if the participant is assigned to the CCBC arm, the Data Manager will notify the site intervention coordinator and pass on the required contact information. For participants in both arms, the Data Manager will also ensure that treating Psychiatrists are provided with the specially designed clinical booklet to record treatment details for the next 12 months.

For statistical reasons (see 'sample size estimation' below) there will be unequal allocation, with a larger number in the intervention arm. The rationale for the unequal randomization is the additional source of variability in the intervention arm - the effect of individual community health workers which is built into the analysis and allowed for in the sample size as recommended in the recent CONSORT guidelines for non pharmacological trial design and reporting[15]. It is difficult to precisely estimate the magnitude of this extra variability (the intra-class correlation), but the value chosen for the intervention arm of 0.1 is conservative: a recent review of a large number of primary care variables found the inter-quartile range to be 0 to 0.032[16]. For practical reasons, the ratio of numbers in the two arms was fixed at a whole number; for the study this has been chosen to be 2:1. The method of allocation will be through the use of permuted blocks, with random block sizes chosen from 3, 6 and 9 using the '*ralloc*' routine in Stata.

##### Outcome assessments

###### 1. Quantitative assessments

For the primary outcome assessments, we will record the *symptoms of schizophrenia *at baseline and at 12 months, with the Positive and Negative Syndrome Scale (PANSS) [17].

For measuring the *change in disability*, we will use the Indian Disability Evaluation and Assessment Scale (IDEAS)[18] at baseline, 6 and 12 months to generate information on the time course of the effect of the intervention.

For the secondary outcome assessment for persons with schizophrenia, we will measure:

• *Adherence with antipsychotic medication*: For each participant, we will assess adherence at baseline (if on treatment) and at 6 and 12 months using a 5 point ordinal scale developed for the study.

• For measuring *various dimensions of the experience of stigma and discrimination*, we will use the Discrimination and Stigma Scale (DISC)[19] at baseline and at 12 months.

• An item on *willingness to disclose mental illness *[20] will be used for participants at baseline and at 12 months to assess their readiness to disclose their illness.

• The Alienation subscale of the Internalized Stigma of Mental Illness (ISMI) scale[21] will be used to measure the *subjective experience of internalized stigma of participants *at baseline and at 12 months.

• Finally, to estimate the *Quality Adjusted Life Years (QALY)*, we will use the widely used Euroqol (EQ-5D) [22] at baseline and at 12 months.

**The secondary outcome measure for the **primary care givers are:

• The *family burden of caring *will be measured at baseline and at 12 months by using the Burden Assessment Schedule (BAS) which has been developed and used widely in India[23].

• The *knowledge and attitudes of family members about schizophrenia *will be assessed at baseline and at 12 months by using the Knowledge about Schizophrenia Interview (KASI) [24]

• A section of the 'Family Interview Schedule' (FIS) developed for the International Study of Schizophrenia (ISOS)[25] and previously used in a study in India[26] will be employed to assess *the experiences of stigma and discrimination experienced by the primary caregivers*.

• The item on *willingness to disclose mental illness *[20]will be used for caregivers after suitable modification.

• The *caregiver summary assessment of the participant's degree of adherence *will be rated by using the same 5 point ordinal scale used for participants. To improve the validity of adherence ratings, we will complete a systematic assessment of medication use for the last 2 months (to minimize recall bias over the 6 month period); this 2 month adherence will be extrapolated for the last 6 months.

For the economic aspects of the study, we will ask caregivers to complete the Cost of Illness Schedule (CIS) that has been developed and used in India in community studies before[27] at baseline, 6 and 12 months. In addition, we will record the type, dosage and duration of medications prescribed by treating Psychiatrists during each of the contacts with participants as well as the number of inpatient days for each participant - this data will be available from the Psychiatrists' structured records. Service costs will be calculated by attaching locally calculated unit costs to these data. In addition, we will use the Euroqol EQ-5D with participants for assessing Quality of Life Years at baseline and at 12 months.

The adherence measures and the CIS will be repeated at 6 months, as will the IDEAS. For the CIS and adherence measures, the rationale is to minimize recall bias. The IDEAS will be used at 6 months to have an interim measure for one of the primary outcomes that is brief and easy to complete.

Table 2 describes the data collection details of the study.

**Table 2:** Summary table of data collection in COPSI.

TYPE OF DATA					
**1. Quantitative**: (using a combination of programmed palmtops and paper and pencil methods)			**Measured at**:		

**Primary Outcome**	**Interviewee**	**Outcome assessment scale description**	**0 month**	**6 months**	**12 months**

Symptoms	Person with schizophrenia	*Positive and Negative Syndrome Scale (PANSS)*. The PANSS has 30 items to record current psychopathology on 3 domains- positive symptoms, negative symptoms and general psychopathology; each item rated on 1-7 scale thus having a total score range of 30-210.	**X**		**X**

Disability	Primary caregivers	*Indian Disability Evaluation and Assessment Scale (IDEAS)*. For the IDEAS evaluation, the participant is assessed on 4 domains- Self Care, Interpersonal Activities, Communication and Understanding and Work. The scale generates a global score of 0-20 with increasing scores indicating more severe disabilities	**X**	**X**	**X**

**Secondary outcomes**					

Stigma and discrimination	Person with schizophrenia	*Discrimination and Stigma Scale (DISC)*. The version to be used is based on DISC-12, but was adapted for the study. The revised version has 30 items in total, all rated on a 4 point Likert scale ranging from 'not at all' to 'a lot'.	**X**		**X**
		There are 20 items in the Negative Discrimination Subscale plus 1 new negative discrimination item generated for the trial, 4 items on anticipated discrimination, 1 item relating to efforts of overcoming stigma and discrimination and 4 items for recording experiences of positive discrimination.			
		
Internalized stigma		*The Internalized Stigma of Mental Illness (ISMI) scale *is designed to measure the subjective experience of internalized stigma and consists of 5 subscales. For the study, the 'Alienation' subscale was chosen. It consists of 6 items which are scored on a 4-point Likert scale ranging from 'strongly disagree' to 'strongly agree'.	**X**		**X**
		
Willingness to disclose illness		An item on willingness to disclose mental illness rated on a 5-point Likert scale ranging from 'very uncomfortable' to 'very comfortable'.	**X**		**X**
		
Quality of life		Euroqol EQ-5D that has a descriptive assessment of 5 domains: mobility, self-care, usual activities, pain/discomfort, anxiety/depression. Each domain is coded as level 1, 2 or 3 and combined into a 5 digit code together with a summary visual analogue scale assessment of quality of life score 0-100.	**X**		**X**
		
Subjective rating of adherence with antipsychotic medication		Adherence rating tool with 5 ordinal ratings to describe a range of adherence (from non adherent- fully adherent)	**X**	**X**	**X**

Knowledge of and attitudes towards illness	Primary caregiver(s)	*Knowledge about Schizophrenia Interview (KASI)*. There are 6 domains of understanding that are assessed; the maximum score for each domain being 4.	**X**		**X**
		
The family burden of caring		*The Burden Assessment Schedule (BAS)*. The BAS is scored on 40 items with a maximum score of 120; the minimum score can be less than 40 as some questions can be rated as 'not applicable'.	**X**		**X**
		
Costs of illness		Cost of Illness Schedule (CIS)	**X**	**X**	**X**
		
Adherence with antipsychotic medication		Adherence rating tool with 5 ordinal ratings ranging from non- adherent to fully adherent supplemented by audit of actual medicine use over last 2 months that is extrapolated to the previous 6 month period	**X**	**X**	**X**
		
Experiences of stigma and discrimination		14 items from the Stigma section of the *Family Interview Schedule (FIS) *will be used to assess the experiences of stigma the primary caregivers. These cover several aspects of internalized stigma as well including perceived need to conceal the illness and anticipated discrimination. The items are scored on a 4-point Likert scale (0 - 3) ranging from 'not at all' to 'a lot'.	**X**		**X**
		
Willingness to disclose		Item on willingness to disclose mental illness rated on a 5-point Likert scale ranging from 'very uncomfortable' to 'very comfortable'.	**X**		**X**

**2. Clinical outcomes of interest**:	**Information source**	**How recorded?**	**Frequency of measurement**		

Treating Psychiatrist's assessment of overall clinical change	Clinical records maintained by treating Psychiatrist	Clinical Global Impression-Schizophrenia 'overall change' scale section	Every 3 months		
					
Treating Psychiatrist's assessment of adherence		5 point nominal measure, similar to that used by participants and caregivers	Every 3 months		
					
Inpatient stay details		Recorded for each such episode by treating Psychiatrist	Collated at endpoint		
					
Relapse of illness		Relapse is defined as clinically significant exacerbations of symptoms after at least 2 months of well- being; clinical significance involves meeting at least 2 of the following 3 criteria: marked increase in positive symptoms, hospitalization for acute care and significant increase in dosage of antipsychotic medications	Collated at endpoint		
					
Serious antipsychotic medication side effects	As above; also during by 6 and 12 month outcome assessments.		Collated at endpoint		
			
**3. Process indicators**					
			
A. For participants in both arms of study	Clinical records maintained by treating Psychiatrist	Number of contacts with treating Psychiatrists Type (face to face or telephone) contacts Treatment details- use of psychotropic medications and Electro Convulsive Treatments			
B. For participants in the CCBC arm	Individual care plan records maintained by the Community Health Workers' (CHW's)	The delivery of the components of the intervention as per protocol over the 3 phases of the intervention	Collated at endpoint		
		Per protocol supervision for CHW's	Every 3 months		
		Total number of contact by the CHW's during the 12 month period of the intervention	Every 3 months		

Finally, we will record other presence of co-morbid mental and physical disorders and relevant socio- demographic information about the participant, the primary caregiver and the family during the baseline assessment.

####### Outcome evaluation/masking

The outcome evaluation of symptoms using the PANSS will be carried out by mental health professionals who will be trained, certified and supervised by experts. All other outcome measurements will be conducted by trained graduate researchers. The selected researchers will be trained in a structured manner across the sites to ensure adequate inter-rater reliability for all scale items across the sites. To maintain blinding of these assessments, all outcome measures will be administered by researchers who are independent of the intervention and blind to the allocation of treatment. Since neither the participant nor the family caregivers will be blind to their allocation status at the time of the 6 and 12 month interviews, we intend to minimize the chances of unmasking during the outcome assessments by instituting the following measures:

• The intervention and research teams at the sites will not have any interactions during the trial with separate physical location and administrative management.

• The researchers are told that they are evaluating two interventions and that there is genuine equipoise about which one is better

• Orient the families prior to each assessment that they should not disclose whether or not they are receiving home visits from the CHW and

• Complete the primary outcome measures (PANSS and the IDEAS) first.

###### 2. Qualitative Data Collection

The purpose of qualitative data collection within the trial is to describe the experiences of participants and caregivers in the two arms relating to the care received (including acceptability and perceived effect), the impact of the illness and experiences of stigma and discrimination. We also intend to describe changes in these domains over the follow-up period, explore attributions of changes and to compare outcomes between the two arms of the trial.

We intend to recruit approximately 36 participants and 36 primary caregivers (= 36 participant - caregiver dyads) at each site for the in-depth-interviews at baseline; however, numbers may be increased if indicated by ongoing analyses. Separate consent will be obtained from participants and caregiver(s) for these qualitative interviews. The same participants and caregivers who are interviewed at baseline will be approached for follow-up interviews 12 months later after the final quantitative outcome assessments are completed.

The sampling strategy is purposive, taking into account participant's gender and the degree to which stigma is a concern to the participant as assessed by quantitative measures. Efforts will be made to purposively interview outliers. Sampling will further aim to ensure sample variability at each research site with regards to severity of illness, highest education levels in the household, type of relationship of caregiver to patient and other themes that may emerge as important from ongoing analyses.

In addition, we propose to conduct in-depth-interviews with Psychiatrists and CHWs at endpoint to ascertain their satisfaction with work, the adequacy of training and supervision and their impressions on the impact of the program.

####### Quality assurance and fidelity management

To ensure that the CCBC intervention is of adequate fidelity, we will collate the intervention process indicators related to the delivery of the intervention (Table 2) every month from each site. This will be monitored to check for significant divergence from per protocol norms between the sites; any such divergence will lead to suitable corrective action to harmonize the intervention across the sites.

For quality assurance purposes, key CCBC sessions at each site (needs assessment, 3 and 6 month reviews and termination session) will be rated by intervention coordinators during joint home visits with the CHWs. Feedback for improving the methods and delivery of the intervention will be provided to all CHWs to achieve and maintain pre determined quality benchmarks during the trial.

Systematic efforts will be made to ensure that both quantitative outcome measurements as well as qualitative interviews will be of adequate quality across the sites. The researchers will be supervised in a weekly group meeting with the study coordinators at each site. In addition, 5% of all interviews at each site will be assessed through joint onsite visits with the coordinator who will provide feedback using a specially designed assessment form to confirm that the overall pre-set quality benchmarks are achieved and maintained during the trial. For the qualitative data, there will be regular supervision available for researchers at each site with regular group and individual supervision related to interview methods, data translation and transcription.

To ensure the safety of the researchers while conducting interviews at the homes of participants, all initial visits will be made by a team of 2 researchers who will have notified the Coordinators about their location. Researchers have also been trained to recognize possible dangers and threats and respond to them appropriately (seating arrangements, non confrontational interview techniques). All researchers will also have access to mobile phones to discuss any potentially difficult situation with their supervisor. In case of any untoward incident, researchers will have provision for debriefing and a review of the safety arrangements.

##### Statistical issues

###### Sample size estimation

Based on the data from the earlier non randomized study[9], it was assumed that a difference of at least 20% reduction on the PANSS total score of 65 (sd 10) i.e. 13 points to 52, would be highly clinically significant. The estimated pre-post correlation was 0.4 (reducing the effective sd to 9 after adjusting for baseline). The intra-class correlation within the three sites in the control arm was set at 0.05; in the intervention arm (which would include within-CHW as well as within-site effects) it was set at a higher level of 0.1; alpha was set as 0.05. Using the method of Roberts and Roberts[28] with the Stata routine *cluspower *will give us 98% power to detect this difference. This is equivalent to a large standardised effect size, 1.44. For effect sizes of 1 (9 PANSS units) and 0.8 (7.2 PANSS units) the power would be approximately 90% and 80% respectively.

The required sample size of 241 was increased to 282 to allow for 15% attrition and rounded up to allow for a 2:1 randomisation within each site. The total number of participants was then divided slightly unequally between sites for reasons of feasibility. A total of 188 persons with schizophrenia will be allocated to the CCBC arm while 94 persons will be allocated to the FBC arm.

##### Data management and analysis

Findings will be reported according to the revised CONSORT guidelines[14]. No interim analyses are planned apart from baseline comparisons. The statistical package Stata will be used for the quantitative analysis. In accordance with good trial practices, data will be retained for 7 years after completion of the trial.

Descriptive summaries of socio-demographic and clinical data will be provided for all trial participants at baseline, and for outcome measures at baseline, 6-month and 12-month follow up points; this will include means and standard deviations, or proportions, in the two arms, as appropriate. Scale and subscale totals with missing items will be pro-rated (i.e. based on the mean of those items that are present) if 20% or fewer items are missing. The PANSS and the IDEAS will be summarised in terms of the total score, the separate sub scores of the different domains and the proportion of patients improving by >20% from baseline. Histograms within each arm will be used to assess normality of the distribution of the data, to identify any outliers and check for data errors. The proportion of participants who are fully, partially or non- adherent to antipsychotic treatment (derived from the adherence ratings of primary caregivers) will be reported for both arms. CHW characteristics such as completed years of education and caseload will also be described.

The data will be analysed under intention-to-treat assumptions (i.e. all those with at least one follow up measurement will be analysed in arms as randomised). The 12-month PANSS total score, adjusted for PANSS scores at baseline and site (using analysis of covariance), and including clustering effects of CHWs, will be used for determining the comparative effects of the two arms on this primary outcome of the study. The disability score generated by the IDEAS will be similarly analysed; in addition, a longitudinal analysis including both 6- and 12-month outcomes will also be performed using a GEE or random effects model including a time x treatment term allowing for modelling changes over time. Residual analysis will be performed to check the distribution of the data and to detect outliers. Logistic regression analysis with >20% reduction in PANSS at 12 months as the dependent variable will be performed. Secondary outcomes at 12 months will be treated in a similar way to the primary outcome; of the secondary outcomes, adherence will be measured at the 6-month point as well as the 12-month point.

A number of sensitivity analyses for the primary outcome are planned to be carried out. Regression models will be re-estimated including possible confounders such as participant characteristics that differ at baseline and may be associated with outcome (e.g. age, gender, duration of illness, socioeconomic status and education) and CHW characteristics like education and caseload; imputation of missing baseline covariates will be performed if there are more than 10% missing and analyses will be repeated.

Subgroup analyses will include site and gender as interactions with treatment arm to estimate differential effects, and further analyses will investigate the effect of engagement with the interventions on outcome. In addition to the primary intention-to-treat analysis, the effect of participants in the CCBC arm receiving the minimum effective number of sessions as defined in the protocol will also be estimated. Power for these subgroup analyses has not been specifically allowed for, and so they will be treated as exploratory.

##### Analysis plan for economic data

Service costs will be calculated by combining the service use data with appropriate local unit costs. Cost comparisons will be made using bootstrapping methods to account for skewed data. Cost-effectiveness will be assessed by combining costs with outcomes in the form of incremental cost-effectiveness ratios which will show the extra cost incurred (if any) to produce a unit improvement in the main outcomes (symptoms and disability) as well as QALYs (measured using the EQ-5D combined with appropriate utility weights). Uncertainty around cost-effectiveness estimates will be explored using cost-effectiveness planes and interpretation will be aided using cost-effectiveness acceptability curves.

##### Analyses plan of qualitative data

Analyses of interview data will commence as data collection is ongoing and will involve importing interview transcripts into qualitative analysis software (NVivo8) for coding and identification of key themes. Analyses of the baseline qualitative data will explore illness experiences of people with schizophrenia and their caregivers, with a particular focus on experiences of stigma and discrimination.

As part of the 12-month follow-up qualitative analyses, treatment arms will be compared with regards to themes such as the impact of the illness on persons with schizophrenia and caregivers including experiences of stigma and discrimination, met and unmet needs and other themes that emerge as important from the data. There will also be a focus on understanding the experiences of care received, including their acceptability and perceived effect, perceptions of interactions with staff and attributions of changes from entry into the trial to endpoint. Examples of possible unintended or even negative consequences of CCBC will be sought out. Staff interviews will concentrate on staff's perspectives on the impact of the intervention on the study outcomes.

##### Trial management and monitoring

The COPSI study will be conducted according to the good clinical practice guidelines recommended for conducting multi site randomized controlled trials. There will be a dedicated Trial Coordinator who will coordinate the recruitment and progress of the trial in conjunction with each of the site Project Management Committees set up for this purpose.

##### Ethical considerations

Even though there is observational evidence from uncontrolled studies about the effectiveness of community based interventions, there is genuine clinical equipoise regarding the question of the effectiveness and affordability of community care in Low and Middle Income Countries (LAMIC). This trial is justified in this context of paucity of evidence from such contexts, derived from rigorous controlled studies, about the clinical and cost effectiveness of community services for people with schizophrenia.

Underpowered trials are unethical and we have made provisions to have adequate power in the COPSI study. We have calculated the sample size in a systematic fashion at several levels. This includes having a threshold of 95% power (using alpha = 0.05) for detecting a clinically significant difference in the primary outcome measure between the two arms. We have also specifically addressed the issue of clustering effects within treatment sites and CHWs delivering the intervention in calculating our sample size. Based on these assumptions, we feel the study is adequately powered to detect differences in a robust manner to generate valid results.

We will ensure that the rights of participants are protected during the conduct of the trial in accordance with good practice ethical obligations. To address the problems associated with obtaining consent from people with schizophrenia who are symptomatic and sometimes non-literate, the consent procedure will be carried out using non technical language and, in a manner designed to enhance the intake and retention of information. This includes the use of a specially designed flipchart illustrating the key components of the information relevant to informed decision making. Irrespective of the decision to participate or not, no person with schizophrenia will be deprived of any treatment s/he will ordinarily receive. Participants will be free to withdraw from the study at any stage without compromising their usual clinical care. Finally, informed consent to participate in the trial will be recorded by using a form that is in compliance with the Helsinki Declaration and Good Clinical Practice (GCP) guidelines[29]; these have been approved by regulatory bodies.

Arrangements will be made to comply with suggested GCP guidelines in related to protecting the confidentiality of personal data principally through procedures to separate study data and participant identifiable data. For the quantitative data, the data gathered in the palmtops for each participant and the family at baseline will be checked by the Data Manager who will remove all personal identification items (to be kept as a separate file with restricted password protected access to the Data Manager only) and assign a unique Trial ID. All future data will be collected and collated using only the ID number. Regarding the qualitative data set, participant and caregiver files will be kept in locked cabinets with only the interviewers and the research coordinator having access. All identifying information (i.e. name, socio-demographic data etc) will be removed from the interview data and kept separately. Both the individuals and caregivers will be given unique ID codes (i.e. not having name/any other identifying information). Soft copies of transcriptions will be kept in password protected files in a secure computer. Transcriptions will have no identifying information.

Formal ethical approval for conducting the trial has been sought and obtained from the ethics committees at the Kings' College, London (PNM/08/09-121), the London School of Hygiene and Tropical Medicine (approval number 5579) and from the respective Institutional Review Boards at SCARF and Sangath. The trial is registered with the International Society for the Registration of Clinical Trials and the allocated unique ID number is ISRCTN 56877013.

The trial will be regulated by an independent Trial Monitoring Committee (TMC) represented by experts and user representatives who have reviewed and formally approved the protocol before commencing recruitment. The TMC will be provided with quarterly updates describing the trial progress during the recruitment period and every 6 months thereafter. A *critical incident register *will be maintained during the study to record four specific serious adverse events- death, suicide attempt, hospitalization (from any cause) and serious medication side effects like neuroleptic malignant syndrome, tardive dyskinesia, akathisia and tremors for participants in both arms of the study; these are in accordance with the recommendations of the TMC. Across both arms, these adverse events will be recorded by the treating Psychiatrists as they occur and by the researchers who will be collecting quantitative data at 6 and 12 months. For participants in the CCBC arm, the concerned CHW will also report these events. The reporting and per protocol action taken for each adverse event will be made available the TMC during the updates. The TMC is also empowered to independently review the ethical and data management procedures of the trial as per established good practice norms.

The TMC can examine the unblinded outcome and adverse event data during the planned annual review meetings and will be consulted before the public reporting of the results of the study. Updates will be provided to all concerned ethics committees of major changes in the protocol, if necessary.

## Discussion

Providing community based services for greater numbers of people with schizophrenia in low and middle income countries is an urgent public health priority. The COPSI randomized controlled trial builds on previous observational evidence of the feasibility, acceptability and effectiveness of CBR programs that incorporated lay community health workers as the front line service providers. This trial is designed to provide quality evidence about the clinical effectiveness of the intervention and the economic implications of the community intervention.

Given the 'real life' sample of subjects and the diversity of the sites where the study will be conducted, we believe that the results of the study will be generalisable beyond the study population. If the results confirm the hypotheses and CCBC intervention has additional clinical benefits and is cost effective, this can have significant impact on health policy related to the scaling up community care for people with schizophrenia in India and other LAMIC. The results of the trial will be used to inform policy makers and practitioners about the benefits, necessary human resources and costs of a community based intervention that can be fronted by non-specialist workers (working in tandem with specialists), involve the person with schizophrenia and the family and engage proactively with the local community in promoting recovery and social inclusion.

Finally, through the detailed exploration of experiences of stigma and discrimination and evaluation of the intervention on stigma outcomes, the study will also contribute to the limited evidence on felt experiences and effective interventions to reduce stigma and discrimination for people with severe mental disorders in LAMIC.

## Competing interests

The authors declare that they have no competing interests.

## Authors' contributions

SC, MD was responsible for the initial drafting of the paper and for coordinating the revisions leading to the final paper for submission. ML, PhD and KG, MSc, MPH provided specific inputs for the statistics section of the paper. MK MRCPsych, is a Wellcome Trust Clinical Research Fellow and provided specific inputs for drafting the qualitative data rationale and analysis plan aspects of the paper. PM, PhD specifically contributed to the drafting of the economic aspects of the paper. SJ, MA, Research Coordinator, SN, B.Sc Research Coordinator, HD, DNB and MB, MA, MSc, and formerly project coordinator, helped with the sections on flow of participants, data management and description of usual care of the paper. MV, MD and Professor, Department of Psychiatry, NIMHANS, RT, MD, PhD, and Director, SCARF, VP, PhD, Professor of International Mental Health & Wellcome Trust Senior Clinical Research Fellow in Tropical Medicine and GT, PhD, Professor and Head of Health Service and Population Research Department, Institute of Psychiatry were closely involved in providing feedback and the overall guidance and mentoring for the drafting of the paper. All authors have read the paper and are in agreement with the contents.

## References

[B1] Thara R Twenty-year course of schizophrenia: the Madras Longitudinal Study Can J Psychiatry 2004 49 8 564 9 15453106 10.1177/070674370404900808

[B2] Kurihara T Eleven-year clinical outcome of schizophrenia in Bali Acta Psychiatr Scand 2005 112 6 456 62 10.1111/j.1600-0447.2005.00604.x 16279875

[B3] WHO The global burden of disease 2004 update 2008 Geneva: World Health Organization

[B4] WHO The World Health Report Mental health: New Understanding, New Hope 2001 Geneva: World Health Organization

[B5] Thornicroft G Tansella M Components of a modern mental health service: a pragmatic balance of community and hospital care: overview of systematic evidence Br J Psychiatry 2004 185 283 90 10.1192/bjp.185.4.283 15458987

[B6] Jacob KS Mental health systems in countries: where are we now? Lancet 2007 370 9592 1061 77 10.1016/S0140-6736(07)61241-0 17804052

[B7] Chisholm D Scale up services for mental disorders:A call to action Lancet 2007 Global Mental Health 87 98 17804059 10.1016/S0140-6736(07)61242-2

[B8] WHO, ILO and UNESCO CBR:A strategy for rehabilitation, equalization of opportunities, poverty reduction and social inclusion of people with disabilities 2004 WHO, Geneva

[B9] Chatterjee S Evaluation of a community-based rehabilitation model for chronic schizophrenia in rural India Br J Psychiatry 2003 182 57 62 10.1192/bjp.182.1.57 12509319

[B10] Chatterjee S Outcomes of people with psychotic disorders in a community-based rehabilitation programme in rural India Br J Psychiatry 2009 195 5 433 9 10.1192/bjp.bp.108.057596 19880934 PMC2806571

[B11] WHO The ICD-10 classification of mental and behavioural disorders- Research and Diagnostic criteria 1992 Geneva: World Health Organization

[B12] Haro JM Kamat SA Ochoa S The Clinical Global Impression-Schizophrenia Scale: a simple instrument to measure the diversity of symptoms present in schizophrenia Acta Psychiatr Scand 2003 107 Suppl (416) 16 23 10.1034/j.1600-0447.107.s416.5.x 12755850

[B13] Carpenter TW Decisional capacity for informed consent in schizophrenia research Archives of general Psychiatry 2000 57 533 538 10.1001/archpsyc.57.6.533 10839330

[B14] Schulz KF CONSORT 2010 statement: updated guidelines for reporting parallel group randomized trials Trials 2010 11 32 10.1186/1745-6215-11-32 20410783

[B15] Boutron I Extending the CONSORT statement to randomized trials of nonpharmacologic treatment: explanation and elaboration Ann Intern Med 2008 148 4 295 309 18283207 10.7326/0003-4819-148-4-200802190-00008

[B16] Adams G Patterns of intra-cluster correlation from primary care research to inform study design and analysis J Clin Epidemiol 2004 57 8 785 94 10.1016/j.jclinepi.2003.12.013 15485730

[B17] Kay SR Fiszbein A Opler LA The positive and negative syndrome scale (PANSS) for schizophrenia Schizophr Bull 1987 13 2 261 76 3616518 10.1093/schbul/13.2.261

[B18] Thara R Indian Disability Evaluation and Assessment Scale 2002 R.C.o.I.P. Society

[B19] Thornicroft G Global pattern of experienced and anticipated discrimination against people with schizophrenia: a cross-sectional survey Lancet 2009 373 9661 408 15 10.1016/S0140-6736(08)61817-6 19162314

[B20] TNS UK for the Care Services Improvement Partnership, D.o.H. Attitudes to mental illness 2009 research report 2009

[B21] Boyd Ritcher J Internalized stigma of mental illness: psychometric properties of a new measure Psychiatric Research 2003 121 31 49 10.1016/j.psychres.2003.08.008 14572622

[B22] Group E EuroQol: a facility for the measurement of health-related quality of life Health Policy 1990 16 199 208 10.1016/0168-8510(90)90421-9 10109801

[B23] Thara R Padmavathi R Kumar S Srinivasan L Burden Assessment schedule: Instrument to assess burden on caregivers of chronic mentally ill Indian Journal of Psychiatry 1998 40 21 29 PMC2964812 21494438

[B24] Barrowclough C Tarrier N Watts S Vaughn C Bamrah JS Freeman HL Assessing the functional value of relatives' knowledge about schizophrenia: a preliminary report British Journal of Psychiatry 1985 151 1 8 10.1192/bjp.151.1.1 3676605

[B25] Sartorius N Gulbinat G Harrison G Long term follow-up of schizophrenia in 16 countries Social Psychiatry &Psychiatric Epidemiology 1996 31 249 258 10.1007/BF00787917 8909114

[B26] Thara R TN S How stigmatising is schizophrenia in India? Int J Soc Psych 2000 46 135 10.1177/002076400004600206 10950361

[B27] Chisholm DSK Kumar KK Saeed K James S Mubbashar M Integration of mental health care into primary care. Demonstration cost-outcome study in India and Pakistan Br J Psychiatry 2000 176 581 588 10.1192/bjp.176.6.581 10974966

[B28] Roberts C Roberts SA Design and analysis of clinical trials with clustering effects due to treatment Clin Trials 2005 2 2 152 62 10.1191/1740774505cn076oa 16279137

[B29] Guideline IHT Guideline for good clinical practice E6 (R1). in International conference on harmonization of technical requirements for registration of pharmaceuticals for human use 1996

